# Association of Environmental Toxicants and Conduct Disorder in U.S. Children: NHANES 2001–2004

**DOI:** 10.1289/ehp.11177

**Published:** 2008-03-11

**Authors:** Joseph M. Braun, Tanya E. Froehlich, Julie L. Daniels, Kim N. Dietrich, Richard Hornung, Peggy Auinger, Bruce P. Lanphear

**Affiliations:** 1 Department of Epidemiology, University of North Carolina, Chapel Hill, Chapel Hill, NC, USA; 2 Division of General and Community Pediatrics, Department of Pediatrics; 3 Cincinnati Children’s Environmental Health Center, Department of Pediatrics and; 4 Division of Developmental and Behavioral Pediatrics, Department of Pediatrics, Cincinnati Children’s Hospital Medical Center, Cincinnati, Ohio, USA; 5 Division of Epidemiology and Biostatistics, The University of Cincinnati College of Medicine and Academic Health Center, Cincinnati, Ohio, USA

**Keywords:** conduct disorder, cotinine, epidemiology, lead poisoning, NHANES, tobacco smoke

## Abstract

**Objective:**

The purpose of this study was to examine the association of tobacco smoke and environmental lead exposure with conduct disorder (CD).

**Methods:**

The National Health and Nutrition Examination Survey (NHANES) 2001–2004 is a nationally representative cross-sectional sample of the noninstitutionalized U.S. population. We examined the association of prenatal tobacco, postnatal tobacco, and environmental lead exposure with CD in children 8–15 years of age (*n* = 3,081). We measured prenatal tobacco exposure by parent report of cigarette use during pregnancy, and postnatal tobacco using serum cotinine levels. We assessed lead exposure using current blood lead concentration. Parents completed the Diagnostic Interview Schedule for Children to determine whether their children met criteria of the *Diagnostic and Statistical Manual of Mental Disorders*, 4th edition (*DSM-IV* ) for CD.

**Results:**

Overall, 2.06% of children met *DSM-IV* criteria for CD in the past year, equivalent to 560,000 U.S. children 8–15 years of age. After adjustment, prenatal tobacco exposure was associated with increased odds for CD [odds ratio (OR) = 3.00; 95% confidence interval (CI), 1.36–6.63]. Increased blood lead levels (fourth vs. first quartile) and serum cotinine levels (fifth vs. first quintile) were associated with an 8.64-fold (95% CI, 1.87–40.04) and 9.15-fold (95% CI, 1.47–6.90) increased odds of meeting *DSM-IV* CD criteria. Increasing serum cotinine levels and blood lead levels were also associated with increased prevalence of CD symptoms (symptom count ratio, lead: 1.73; 95% CI, 1.23–2.43; symptom count ratio, cotinine: 1.97; 95% CI, 1.15–3.40).

**Conclusions:**

These results suggest that prenatal tobacco exposure and environmental lead exposure contribute substantially to CD in U.S. children.

Conduct disorder (CD) is characterized by persistent behavioral patterns that violate social rules and the rights of individuals (American Psychiatric Association 2000). Children with CD display aggression toward people and animals, intentionally destroy others’ property, and chronically steal or deceive. Children with CD are at increased risk for drug and alcohol abuse, antisocial personality disorder, and anxiety-related disorders (Goldstein et al. 2006; Gunter et al. 2006). Public expenditures related to general and mental health care, school services, and juvenile justice for children with CD exceed $10,000 per child per year (Foster and Jones 2005).

The national prevalence of CD is unknown. It is estimated from community-based regional samples that 0.4–3.3% of children and adolescents have CD, with males two to three times more likely than females to receive a CD diagnosis (Lahey et al. 2000; Loeber et al. 2000; Maughan et al. 2004). Prior variation in CD prevalence estimates may be related to the informant, the diagnostic instrument, the version of *Diagnostic and Statistical Manual of Mental Disorders* (*DSM*) used (3rd ed., 3rd ed. rev., or 4th ed.), participants’ age, socioeconomic status, and degree of urbanicity (Loeber et al. 2000). To date, no studies have provided an estimate of the national prevalence of CD in U.S. children using criteria of the 4th edition of the *DSM* (*DSM-IV*; American Psychiatric Association 2000).

Questions also persist about the underlying risk factors for the development of CD (Burke et al. 2002). Prenatal tobacco smoke exposure has been consistently associated with disruptive behavior disorders, such as oppositional defiant disorder and CD, even after controlling for potential confounders, including sociodemographic factors, prenatal insults, and parental psychopathology (Fergusson et al. 1993, 1998; Wakschlag et al. 1997, 2002; Weissman et al. 1999). Results from a prospective cohort in New Zealand indicate that children exposed to tobacco smoke *in utero* had CD symptom rates approximately two times higher than unexposed children (Fergusson et al. 1998). Another prospective study of approximately 6,000 Finnish males found that prenatal tobacco smoke exposure was associated with a 1.74-fold increased odds of committing a delinquent act in late childhood or early adulthood (Rantakallio et al. 1992).

Although prior studies have documented associations between prenatal tobacco smoke exposure and CD, the relationship between postnatal environmental tobacco smoke (ETS) exposure and CD is less clear (Fergusson et al. 1998; Weitzman et al. 1992). Weitzman et al. (1992) reported significant increases in the number of behavior problems among children whose mothers smoked only after pregnancy. Compared with children of nonsmoking women, children exposed to postnatal ETS had a 2-fold increased odds of having extreme behavior problems on the Behavior Problem Index of the Child Behavior Checklist (CBCL) (Weitzman et al. 1992). Similarly, in a New Zealand cohort, Fergusson et al. (1998) found an increased number of CD symptoms among children exposed to post-natal ETS. To date, none of these studies has used a biomarker of tobacco smoke exposure to measure the association between ETS and behavior problems. This limitation can result in exposure misclassification, because a substantial proportion of women who report no ETS exposure have measurable cotinine levels (DeLorenze et al. 2002).

The relationship between environmental lead exposure and violent, aggressive, and oppositional behavior was first reported by Byers and Lord (1943). They observed that among 20 lead-poisoned children, 19 who had “recovered” from lead poisoning failed high school or had behavioral problems. Since then, elevated bone and blood lead levels have been associated with an increased risk of juvenile delinquency in late childhood and early adulthood in case–control and prospective cohort studies (Dietrich et al. 2001; Needleman et al. 1996, 2002). Earlier studies observed children with higher blood lead levels than the levels currently seen; thus, inferences may not be directly relevant to contemporary children with lower blood lead levels (Dietrich et al. 2001; Needleman et al. 1996, 2002). Additionally, recent studies have observed associations between blood lead levels below the current Centers for Disease Control and Prevention recommended action level of 10 μg/dL and cognitive and behavioral deficits (Braun et al. 2006; Lanphear et al. 2005). It is unclear whether lower blood lead levels are associated with more severe behavioral problems such as CD in children.

The purpose of this study was to provide an estimate of the national prevalence of CD defined using *DSM-IV* criteria and to test the hypothesis that exposures to ETS and childhood lead exposure were associated with CD in a large nationally representative sample of U.S. children.

## Materials and Methods

### Data source

The data for this analysis came from the National Health and Nutrition Examination Survey (NHANES), conducted from 2001 through 2004. NHANES is a cross-sectional household survey of the non-institutionalized civilian population. NHANES used a complex, multistage probability sampling design, with oversampling of adolescents 12–19 years of age, adults ≥ 60 years of age, low-income persons, Mexican Americans, and non-Hispanic blacks. This method of over-sampling allows for more valid and precise estimates to be derived among subgroups than a simple random sampling methodology would. Details regarding interviews, examination procedures, and sample collection have been described elsewhere (National Center for Health Statistics 2007b).

### Assessment of CD

The National Institute of Mental Health’s Diagnostic Interview Schedule for Children–Caregiver Module (DISC) was used to assess for the presence of mental health disorders based on *DSM-IV* criteria. The DISC is a structured diagnostic interview instrument designed for use by lay interviewers in clinical and epidemiologic studies. Reliable versions are available in English and Spanish (Bravo et al. 2001; Shaffer et al. 2000). Caregivers of children 8–15 years of age completed the CD DISC module in English or Spanish by phone 2–4 weeks after the child’s NHANES Mobile Examination Center (MEC) visit, providing information about the child’s CD symptoms over the preceding 4 weeks, 12 months, and lifetime. DISC algorithms were used to determine whether the child met the criteria for CD diagnosis within the last 4 weeks, 12 months, and lifetime. For this analysis, our primary outcomes were meeting *DSM-IV* CD criteria (dichotomous) and CD symptom count in the preceding 12 months. Children met *DSM-IV* CD criteria if they had three or more symptoms in the preceding 12 months, with at least one symptom in the preceding 6 months. Symptom counts during the preceding 12 months for children 8–15 years of age ranged from 0 to 12 symptoms.

### Environmental exposures

We used parent report to measure children’s exposure to tobacco products. Measurement of prenatal tobacco smoke exposure consisted of the question, “Did the child’s biological mother smoke at any time while she was pregnant with him/her?” No information on the quantity or brand of cigarettes smoked during pregnancy was collected.

Information on postnatal ETS exposure included parent report about the presence of a smoker in the home, the number of packs of cigarettes smoked in the home, and the child’s serum cotinine levels. Exposure to household ETS was assessed by asking, “Does anyone who lives here smoke cigarettes, cigars, or pipes anywhere inside this home?” In addition, interviewers asked for the number of cigarettes smoked per day inside the home. Cotinine, a metabolite of nicotine, was measured using high-performance liquid chromatography–tandem mass spectrometry (Bernert et al. 2000). The limit of detection (LOD) for this assay was 0.015 ng/mL; 573 (20.0%) children had levels below this level. Cotinine levels > 10 ng/mL (*n* = 82) are indicative of active smoking; thus, children with values above this level were excluded from all analyses (Benowitz et al. 1983).

Blood lead concentration was determined by graphite furnace atomic absorption spectrophotometry (Miller et al. 1987; Parsons and Slavin 1993). The LOD was 0.3 μg/dL; 38 children had blood lead levels below this threshold. We ran secondary analyses excluding children with blood lead levels ≥ 10 μg/dL (*n* = 6) to determine whether they had excessive influence on our models.

### Covariates

We examined covariates thought *a priori* to possibly confound the relationship between CD and prenatal tobacco smoke, postnatal ETS, and lead exposure. Demographic variables included the child’s age, sex, race, socioeconomic status as measured by poverty-to-income ratio (PIR), and mother’s age at child’s birth (< 18 years vs. ≥ 18 years). PIR is the ratio of household income to the poverty threshold for a family of a given size in the respective year of the interview. PIR provides for a better measure of socioeconomic status by controlling for both income and household size. PIR values were coded into four categories to reflect the current standards used in government-financed welfare programs (< 1.00, 1.00–1.85, 1.85–3.0, and > 3.0). Because low birth weight and neonatal intensive care unit (NICU) admission may act as intervening variables on the causal pathway between prenatal tobacco smoke exposure and CD, we did not include these two variables in our multivariate analyses.

### Statistical analysis

We used logistic regression to analyze the associations among environmental, demographic, and medical factors with meeting *DSM-IV* CD criteria in the past year (yes/no). We used Poisson regression to analyze the association between children’s symptom count in the preceding year and demographic, medical, and environmental factors. We used Poisson regression models to compare the ratio of symptom counts across demographic, medical, and environmental variables along with respective mean symptom counts within strata of these variables. We did not use an offset term in our Poisson models.

Because cotinine provides a more objective measure of ETS exposure than parent report, we used it in our primary analyses. Serum cotinine and blood lead levels were categorized into quintiles and quartiles, respectively, using weighted percentages.

We conducted a secondary analysis to determine the effect of postnatal ETS exposure among children without prenatal tobacco smoke exposure. To provide more precise estimates of the effect, we conducted this analysis using only CD symptom counts (Poisson models).

We used logistic regression models to calculate the odds of meeting *DSM-IV* CD criteria within each quintile of cotinine exposure or quartile of lead exposure, using the first quintile or quartile as the common referent group. We fit Poisson regression models with the same quintile and quartile categorical variables that we used in logistic regression models.

Regression diagnostics identified influential observations, overdispersion, or collinearity, and we observed no collinearity or overdispersion. Potential influential observations were identified in the logistic regression models (*n* = 4) and Poisson regression models (*n* = 20) using standardized residuals. The exclusion of these outliers did not appreciably alter the estimates of prenatal tobacco smoke exposure, postnatal ETS exposure, or blood lead levels. We report here all multivariable results with influential observations.

We performed analyses using the SUDAAN statistical package to account for the multistage, complex sampling design (Research Triangle Institute 2004). Sample weights were applied according to the National Center for Health Statistics guidelines to produce accurate national estimates, adjusting for the oversampling of minorities and young children (National Center for Health Statistics 2007a).

The institutional review boards of the National Center for Health Statistics and the University of North Carolina, Chapel Hill, approved this study. Informed consent was obtained from all participants (National Center for Health Statistics 2007b).

## Results

A total of 3,907 children 8–15 years of age were interviewed, and 3,799 (97.2%) of these children completed the MEC examination in 2001–2004. A total of 3,081 (78.9%) parents completed the DISC telephone interview, and 2,619 (67.0%) children had complete data available for multivariable analysis. Table 1 lists the characteristics of children whose parents did and did not complete the DISC telephone interview. Children whose parents completed the DISC were more likely to be older (13–15 years of age), white, and in the highest PIR category, to have lower blood lead levels, to not live with a smoker, and to have a birth weight > 2,500 g, compared with the 826 (21.1%) children whose parents did not complete the DISC.

Of the 3,081 children 8–15 years of age available for analysis, 68 [2.06%; 95% confidence interval (CI), 1.47–2.88%] met the *DSM-IV* criteria for CD in the past year, equivalent to 560,000 U.S. children and adolescents. Children exposed to prenatal tobacco smoke, postnatal ETS, and environmental lead had a higher prevalence of CD. CD prevalence was higher among male children (2.24%; 95% CI, 1.66–3.02%) than female children (1.86%; 95% CI, 1.11–3.12%). The prevalence of CD was also higher among children 13–15 years of age than among children < 13 years of age.

The mean CD symptom count (Table 2) for all U.S. children 8–15 years of age was 0.60 (95% CI, 0.54–0.67). Children who met *DSM-IV* criteria for CD had, on average, 6.26 (95% CI, 5.70–6.83) symptoms in the past year, whereas children without CD had 0.49 (95% CI, 0.44–0.53) symptoms. Mean symptom counts were higher among male children, older children, and those exposed to environmental toxicants (Table 2).

Among children with cotinine levels ≤ 10 ng/mL, 79.1% had detectable cotinine levels. Prenatal tobacco smoke exposure was moderately correlated with the presence of a smoker in the home and the number of cigarettes smoked in the home (Spearman rank *r* = 0.36–0.38). Serum cotinine levels were moderately correlated (Spearman rank *r* = 0.31) with prenatal tobacco smoke exposure and highly correlated with self-reported tobacco exposures (Spearman rank *r* = 0.60). All children (100%) living with a smoker in the home had detectable serum cotinine levels (> 0.015 ng/mL). Among children whose parents did not report a smoker in the home, 74.0% had detectable cotinine levels.

In multivariable analyses, both prenatal and postnatal exposure to tobacco smoke and children’s blood lead levels were significantly associated with meeting *DSM-IV* criteria for CD when adjusted for covariates (Table 3). Children exposed to prenatal tobacco smoke had a 3.00-fold higher odds of CD than did nonexposed children (95% CI, 1.36–6.63). An increase in blood lead levels (fourth vs. first quartile) was associated with an 8.64-fold (95% CI, 1.87–40.04) increased odds of meeting *DSM-IV* CD criteria. Children with serum cotinine levels in the fifth quintile had a 9.15-fold (95% CI, 1.47–56.90) increased odds of meeting *DSM-IV* CD criteria compared with children with serum cotinine levels below the LOD (0.015 ng/mL).

Poisson regression models showed that exposure to tobacco smoke and children’s blood lead concentrations were associated with increased number of CD symptoms among children. Children with prenatal tobacco smoke exposure had 1.68 (95% CI, 1.14–2.48) times as many CD symptoms as did unexposed children (Table 4). Children with higher serum cotinine levels showed an increasing number of CD symptoms compared with children with nondetectable cotinine levels. Children with higher blood lead levels also showed an elevated number of CD symptoms compared with children in the lowest blood lead quartile.

**Figure 1:**
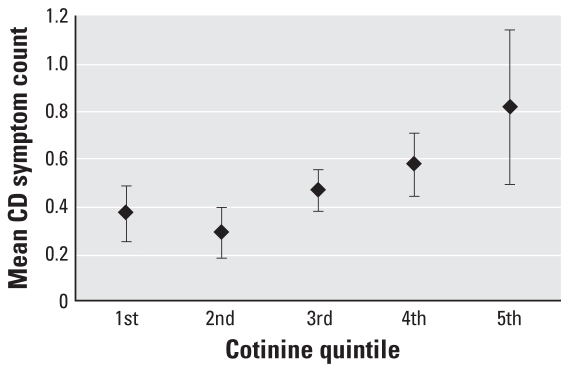
Adjusted mean symptom CD symptom counts by serum cotinine quintile among children without prenatal tobacco smoke exposure, adjusted for child’s age in years, PIR, maternal age at child’s birth, child’s sex, child’s race, prenatal tobacco smoke exposure, and cotinine levels. The mean symptom count is derived from the Poisson regression models presented in Table 4. Error bars represent 95% CIs.

Figure 1 shows the mean number of symptoms among children without any prenatal tobacco smoke exposure. Children with serum cotinine levels in the fifth quintile had 2.23 times (95% CI, 1.21, 4.11) as many symptoms as did children in the first quintile of cotinine exposure. The exclusion of children with blood lead levels ≥ 10 μg/dL did not substantially alter the effect estimates of environmental lead exposure in either our logistic or Poisson regression models.

## Discussion

Overall, 2.06% of children surveyed met *DSM-IV* criteria for CD in the past 12 months, equivalent to 560,000 U.S. children 8–15 years of age. This estimate is consistent with previous prevalence estimates that range from < 1.0% to 16.0% (Lahey et al. 2000; Loeber et al. 2000; Maughan et al. 2004). Our analyses confirm prior observations that prenatal tobacco smoke exposure is associated with disruptive behavior disorders in children. We also found increases in the number of CD symptoms among children exposed to postnatal ETS. Finally, we found that lead exposure, measured using blood lead levels, was associated with increased odds of CD and increased CD symptom count in the past year.

Our results are the first to use a *DSM-IV*–based instrument to assess conduct problems in a nationally representative sample of U.S. children. Previous work evaluated small to moderately sized case–control sets or prospective cohorts using behavior scales such as the CBCL (Needleman et al. 1996; Wasserman et al. 2001), self- or parent report of delinquent behavior (Dietrich et al. 2001; Needleman et al. 1996), or adjudicated case status (Needleman et al. 2002). Estimates of the prevalence of CD may differ across studies because of variations in diagnostic instrument, informant, time period for assessing psychiatric status, and source population. As noted by Lahey et al. (1999), small changes in the diagnostic instrument can produce large changes in the prevalence. When criteria of the 3rd, revised edition of the *DSM* were used, the prevalence of CD in three U.S.-based samples ranged from 1.2% to 16.0%, depending on the age and sex of the children. When *DSM-IV* criteria were used, the prevalence of CD has been reported to be 1.3% for girls and 3.9% for boys (Loeber et al. 2000). We did not find differences in the prevalence of CD diagnosis between boys (2.24%) and girls (1.86%) to be as large as previously reported (Loeber et al. 2000). This may be a result of using parents as the informants of CD symptoms.

In this sample, children with prenatal tobacco smoke exposure had elevated odds of meeting *DSM-IV* CD criteria, which is consistent with previous reports (Fergusson et al. 1998; Wakschlag et al. 1997; Weissman et al. 1999). Using *DSM-IV* criteria, Fergusson et al. (1998) reported a 1.4- to 2.5-fold increase in CD symptom rate among children whose mothers smoked more than one pack of cigarettes per day during pregnancy, which is consistent with the increase in CD symptoms we observed among children exposed to prenatal tobacco smoke. However, unlike our study, Fergusson et al. (1998) did not find an association between maternal smoking during pregnancy and CD diagnosis after controlling for confounding. The difference in our results may be attributable to differences in the confounders that were controlled for. Fergusson et al. (1998) controlled for illicit drug and alcohol use during pregnancy, child-rearing practices, and family functioning. Our reported effect estimates may have been attenuated had we been able to adjust for these other confounders.

Children with increasing cotinine levels had increased odds of meeting *DSM-IV* CD criteria and an increased prevalence of CD symptoms. This association was not a result of increased serum cotinine levels among actively smoking children because we excluded all children with serum cotinine levels indicative of active smoking (≥ 10 ng/mL). Our result is consistent with previous prospective cohort studies that have found similar increases in behavior problems (Weitzman et al. 1992) and CD (Fergusson et al. 1993) associated with postnatal ETS exposure. Fergusson et al. (1998) reported that postnatal ETS exposure was associated with increased CD symptoms at 8, 10, and 12 years of age, using parent informants. Weitzman et al. (1992) reported increased scores on the Behavior Problem Index of the CBCL among children whose mothers smoked only after pregnancy. Our study is the first to use an objective biomarker of tobacco smoke exposure to examine the association between postnatal ETS exposure and severe behavior problems among children.

Our results indicate that a substantial proportion of children are exposed to ETS outside of the home, leading to elevated serum cotinine levels. Previous studies have reported similar findings (Boyaci et al. 2006; Cornelius et al. 2003). Future studies would be well advised to use cotinine as a measure of ETS exposure in children given the high likelihood of exposure misclassification.

Children with blood lead levels ≥ 1.5 μg/dL had a 8.64-fold increased odds of having met *DSM-IV* CD criteria in the past year compared with children with levels from 0.2 to 0.7 μg/dL. Our findings, which are consistent with prior research showing an increased risk of delinquency and criminality among children with higher bone or blood lead levels (Dietrich et al. 2001; Needleman et al. 1996, 2002; Wasserman et al. 2001), provide evidence that contemporary children with considerably lower levels of lead exposure than those in previous studies remain at increased risk for CD. However, the results of our logistic regression models were very imprecise because of the small number of cases.

The relationship between environmental toxicant exposure and disruptive behavior disorders is not surprising given the wealth of animal literature showing adverse effects of nicotine and lead exposure on behavior (Ernst et al. 2001; Lidsky and Schneider 2003). It has been hypothesized that tobacco smoke exposure elicits its neurotoxic effects through two mechanisms: *a*) fetal hypoxia as a result of carbon monoxide exposure and *b*) the direct interaction of nicotine with the developing brain (Wakschlag et al. 2002). Exposure to lead has been observed to cause changes in neurotransmitter concentrations and neurotransmitter receptor density (Lidsky and Schneider 2003). Nicotine interacts with nicotinic acetylcholine receptors, which are present in the developing fetal brain. These receptors are involved in the modulation of neurotransmitters such as dopamine, serotonin, and γ-amino butyric acid. Thus, prenatal nicotine exposure may produce secondary effects through these other systems (Ernst et al. 2001). Slotkin et al. (1987) proposed that nicotine exposure is associated with regional abnormalities in cell number and macromolecule content in rats. In addition, he found that prenatal exposure to nicotine results in a premature switch from cell replication to cell differentiation. Animal models using rats and rhesus monkeys have shown that lead-exposed animals exhibit deficits in discrimination reversal, spatial delayed alternation, and fixed interval tasks (Rice 1996, 2000). These deficits indicate impairment in the animals’ ability to inhibit inappropriate responses, temporally organize behavior, and learn from the consequences of previous actions. Recent work by Nigg et al. (2008) among children with attention-deficit/hyperactivity disorder suggests that behavioral problems may be mediated by child IQ or poor cognitive control.

This study has several limitations that should be considered when interpreting our results. First, the cross-sectional nature of the data makes it difficult to infer causal relationships. The results of our study are consistent with previous birth cohorts that prospectively collected exposure information (Dietrich et al. 2001; Fergusson et al. 1998; Weitzman et al. 1992). Concurrent blood lead levels may not be the optimal biomarker of a child’s risk for lead-associated behavior problems if lead induces neurotoxic effects during early development. However, recent studies indicate that concurrent blood lead level is a stronger predictor of lead-associated IQ decrements and behavior problems than is blood lead measured during early childhood (Chen et al. 2005, 2007; Lanphear et al. 2005). Another potential source of bias in cross-sectional data is exposure misclassification. Mothers of children with behavior problems may be more likely to recall gestational intake of potentially harmful substances, such as tobacco, owing to a drive to identify a cause of their child’s disorder. On the other hand, mothers may fail to report prenatal and postnatal tobacco smoke exposure because of social stigma (i.e., social desirability bias) or tobacco smoke exposures outside of the home. We minimized the possibility that postnatal ETS exposures were misclassified by using cotinine as a marker of exposure. Prior research indicates that mothers can accurately recall gestation intake of tobacco with sensitivities and specificities in the range of 0.81–0.86 and 0.94–0.97, respectively (Jacobson et al. 2002; Tomeo et al. 1999).

Another limitation to the NHANES data is that prenatal tobacco smoke exposure was collected only as a dichotomous variable, and we were unable to examine its relationship with CD in more than two categories. This would result in our effect estimate being biased toward the null if the effect of prenatal tobacco smoke exposure on CD diagnosis is greater at higher levels of prenatal tobacco consumption.

Although we were able to adjust for some confounders, we were unable to adjust for maternal education, family functioning, care-giving environment, parenting practices, prenatal alcohol use, and parental psychopathology. These factors tend to be associated with greater exposure to environmental toxicants and greater risk for CD. NHANES does collect data on maternal education and prena-tal alcohol use, but these data were not available in the publicuse NHANES data files. We did attempt to control for many confounders (or their proxies) in the relationship between environmental toxicants and CD, including race/ethnicity, maternal age at child’s birth, and socioeconomic status (PIR). Still, it is unlikely that these confounders would have been strong enough to eliminate our observed associations, given the previous literature showing robust effects even after controlling for numerous confounders.

Finally, the small number of exposed cases in our logistic regression model created imprecise estimates of the effect of lead exposure and limited our ability to adequately assess for an interaction between prenatal tobacco smoke exposure and blood lead levels. Still, the consistency of our results using the CD symptom counts suggests that exposure to environmental toxins may result in a shift of the symptom count distribution that would result in an increased number of CD-diagnosed children.

The reported prevalence of CD may be an underestimate of the true national prevalence because we relied on parents as the informants of CD symptoms (Loeber et al. 2000; Shaffer et al. 2000). Many disruptive or delinquent behaviors in children are not recognized by their parents. Although maternal and child report of CD symptoms are correlated (Burt et al. 2005), some studies found that child informants were twice as likely to meet the diagnostic criteria for CD compared with parent or caregiver informants (Ezpeleta et al. 1997).

This study confirms the previously observed associations between prenatal tobacco smoke exposure and CD. In addition, this study provides support that elevated blood lead levels are a risk factor for CD. Future research should be directed at confirming this observation in prospective birth cohorts, preferably using serial biomarkers of prenatal tobacco smoke exposure and environmental lead exposure. Despite dramatic reductions in children’s exposures to tobacco smoke and environmental lead, these results suggest that millions of contemporary children may be exposed to levels of these toxicants sufficient to increase the risk for persistent, disruptive, and even violent behavior problems.

## Correction

In Tables 3 and 4, the value for Age in Total no. of cases was incorrect in the manuscript originally published online; it has been corrected here.

## Figures and Tables

**Table 1:** Demographic, environmental, and medical factors by DISC response status [*n* (%)].

Variable	All children (*n* = 3,907)	Children with DISC (*n* = 3,081)	Children without DISC (*n* = 826)
Age (years)			
8–10	1,129 (28.9)	861 (27.9)	268 (32.5)
11–12	982 (25.1)	774 (25.1)	208 (25.1)
13–15	1,796 (46.0)	1,446 (46.9)	350 (42.4)
Missing/refused	0	0	0
Sex			
Male	1,917 (49.1)	1,512 (49.1)	405 (49.2)
Female	1,990 (50.9)	1,569 (50.9)	421 (50.8)
Missing/refused	0	0	0
Race			
Mexican	1,154 (29.5)	928 (30.1)	226 (27.4)
Other Hispanic	150 (3.8)	115 (3.7)	35 (4.3)
White	1,109 (28.4)	905 (29.4)	204 (24.8)
Black	1,331 (34.1)	1,026 (33.3)	305 (36.8)
Other	163 (4.2)	107 (3.5)	56 (6.8)
Missing/refused	0	0	0
PIR			
< 1.00	1,151 (31.1)	875 (29.5)	276 (37.2)
1.00–1.85	892 (24.1)	712 (24.0)	180 (24.3)
1.85–3.00	699 (18.9)	571 (19.3)	128 (17.3)
> 3.00	964 (26.0)	806 (27.2)	158 (21.2)
Missing/refused	201	117	84
Prenatal tobacco exposure			
No	3,258 (84.4)	2,576 (84.6)	682 (82.6)
Yes	601 (15.6)	470 (15.4)	131 (15.9)
Missing/refused	48	35	13
Does anyone smoke in the home?			
No	3,007 (78.0)	2,420 (79.2)	587 (73.2)
Yes	849 (22.0)	634 (20.8)	215 (26.8)
Missing/refused	51	27	24
Cotinine level			
First quintile (< 0.015 ng/mL)	573 (17.5)	509 (18.5)	64 (12.1)
Second quintile (0.015–0.037 ng/mL)	673 (20.5)	583 (21.3)	90 (16.9)
Third quintile (0.038–0.126 ng/mL)	664 (20.3)	568 (20.7)	96 (16.3)
Fourth quintile (0.127–0.673 ng/mL)	766 (23.4)	622 (22.7)	144 (25.0)
Fifth quintile (0.674–9.9 ng/mL)	601 (18.3)	464 (16.8)	137 (29.7)
Active smokers (> 10 ng/mL)	82	64	16
Missing/refused	548	272	276
Blood lead quartiles			
First quartile (0.2–0.7 μg/dL)	746 (22.8)	655 (22.9)	91 (16.3)
Second quartile (0.8–1.0 μg/dL)	800 (23.4)	684 (23.9)	116 (20.7)
Third quartile (1.1–1.4 μg/dL)	746 (21.8)	630 (22.0)	116 (20.7)
Fourth quartile (> 1.5 μg/dL)	1,135 (33.0)	898 (31.2)	238 (42.3)
Missing/refused	480	215	265
Maternal age at birth (years)			
> 18	3,463 (88.6)	2,738 (88.9)	725 (87.8)
≤ 18	444 (11.4)	343 (11.1)	101 (12.2)
Missing/refused	0	0	0
NICU admission			
No	3,393 (87.7)	2,682 (88.8)	711 (86.1)
Yes	476 (12.3)	372 (12.2)	104 (12.6)
Missing/refused	38	27	11
Low birth weight (g)			
≥ 2,500	3,500 (90.9)	2,777 (91.3)	723 (89.6)
< 2,500	350 (9.1)	266 (8.7)	84 (10.4)
Missing/refused	57	38	19

Missing values are not included in percentages.

**Table 2:** Prevalence of CD^a^ and mean DISC symptom count among U.S. children 8–15 years of age according to sociodemographic characteristics and medical and environmental factors.

Variable	No. of cases	Total no.	Weighted percent with *DSM-IV*–diagnosed CD in the past 12 months (95% CI)	Mean symptom count in past year (95% CI)
Total	68	3,082	2.06 (1.47–2.88)	0.60 (0.54–0.67)
Age (years)				
8–10	20	862	1.74 (0.99–3.03)	0.50 (0.41–0.59)
11–12	16	774	1.43 (0.67–3.04)	0.51 (0.36–0.66)
13–15	32	1,446	2.75 (1.71–4.40)	0.76 (0.64–0.88)
Sex				
Male	37	1,513	2.24 (1.66–3.02)	0.70 (0.65–0.75)
Female	31	1,569	1.86 (1.11–3.12)	0.50 (0.39–0.61)
Race				
Mexican	11	928	1.36 (0.62–2.96)	0.47 (0.39–0.54)
Other Hispanic	3	115	2.68 (0.56–11.83)	0.43 (0.15–0.71)
White	19	906	1.94 (1.18–3.19)	0.59 (0.50–0.68)
Black	31	1,026	2.98 (1.76–4.99)	0.89 (0.77–1.00)
Other	4	107	1.75 (0.54–5.52)	0.49 (0.24–0.74)
PIR				
< 1.00	25	875	2.35 (1.22–4.47)	0.78 (0.65–0.91)
1.00–1.85	21	712	3.19 (1.52–6.58)	0.69 (0.53–0.85)
1.85–3.00	8	571	2.10 (0.92–4.70)	0.59 (0.44–0.73)
> 3.00	11	807	1.21 (0.56–2.58)	0.47 (0.37–0.57)
Prenatal tobacco exposure				
No	40	2,577	1.16 (0.79–1.69)	0.48 (0.42–0.55)
Yes	25	470	5.36 (2.98–9.46)	1.09 (0.83–1.35)
Does anyone smoke in the home				
No	38	2,421	1.43 (0.91–2.26)	0.50 (0.44–0.57)
Yes	28	634	4.19 (2.36–7.33)	0.95 (0.81–1.10)
Cotinine level				
First quintile (< 0.015 ng/mL)	3	509	0.31 (0.09–1.01)	0.34 (0.24–0.44)
Second quintile (0.015–0.037 ng/mL)	4	582	0.70 (0.18–2.64)	0.31 (0.19–0.43)
Third quintile (0.038–0.126 ng/mL)	9	568	1.09 (0.40–2.94)	0.54 (0.42–0.66)
Fourth quintile (0.127–0.673 ng/mL)	17	622	4.00 (1.65–9.35)	0.77 (0.59–0.95)
Fifth quintile (0.674–9.9 ng/mL)	26	464	5.15 (2.72–9.52)	1.10 (0.89–1.31)
Blood lead quartiles				
First quartile (0.2–0.7 μg/dL)	4	655	0.32 (0.09–1.06)	0.36 (0.28–0.44)
Second quartile (0.8–1.0 μg/dL)	11	684	1.88 (0.94–3.71)	0.60 (0.47–0.74)
Third quartile (1.1–1.4 μg/dL)	22	630	4.06 (0.94–3.71)	0.70 (0.53–0.86)
Fourth quartile (≥ 1.5 μg/dL)	29	898	3.02 (1.96–4.63)	0.85 (0.74–0.97)
Maternal age at birth of child (years)				
> 18	54	2,739	1.92 (1.30–2.82)	0.58 (0.51–0.65)
≤ 18	14	343	4.02 (1.97–8.03)	0.98 (0.72–1.24)
NICU admission				
No	51	2,683	1.75 (1.13–2.70)	0.57 (0.50–0.65)
Yes	12	372	3.88 (1.98–7.47)	0.77 (0.57–0.97)
Low birth weight (g)				
≥ 2,500	57	2,778	1.88 (1.25–2.80)	0.59 (0.52–0.66)
< 2,500	9	266	4.26 (1.70–10.24)	0.77 (0.50–1.04)

a Diagnosed according to *DSM-IV* criteria using the DISC Caregiver Module.

**Table 3:** Adjusted OR for meeting *DSM-IV* CD diagnosis criteria in the past year among U.S. children 8–15 years of age.^a^

Variable	No. of cases	Total no.	Adjusted OR for meeting *DSM-IV* CD criteria (95% CI)
Age (years)	68	3,082	1.08 (0.88–1.31)
Sex			
Female	31	1,569	Referent
Male	37	1,513	1.00 (0.55–1.80)
PIR			
> 3.00	11	807	Referent
< 1.00	25	875	0.84 (0.26–5.83)
1.00–1.85	21	712	1.24 (0.26–5.83)
1.85–3.00	8	571	1.26 (0.46–3.52)
Race			
White	19	906	Referent
Mexican	11	928	1.01 (0.24–4.27)
Other Hispanic	3	115	0.98 (0.14–7.03)
Black	31	1,026	1.06 (0.42–2.70)
Other	4	107	1.14 (0.27–4.79)
Maternal age at birth of child (years)			
> 18	54	2,739	Referent
≤ 18	14	343	1.30 (0.44–3.87)
Prenatal tobacco exposure			
No	40	2,577	Referent
Yes	25	470	3.00 (1.36–6.63)
Cotinine level			
First quintile (< 0.015 ng/mL)	3	509	Referent
Second quintile (0.015–0.037 ng/mL)	4	582	1.00 (0.15–6.91)
Third quintile (0.038–0.126 ng/mL)	9	568	2.84 (0.46–17.42)
Fourth quintile (0.127–0.673 ng/mL)	17	622	10.22 (1.70–61.40)
Fifth quintile (0.674–9.9 ng/mL)	26	464	9.15 (1.47–56.90)
Blood lead quartiles			
First quartile (0.2–0.7 μg/dL)	4	655	Referent
Second quartile (0.8–1.0 μg/dL)	11	684	7.24 (1.06–49.47)
Third quartile (1.1–1.4 μg/dL)	22	630	12.37 (2.37–64.56)
Fourth quartile (1.5–10.0 μg/dL)	29	898	8.64 (1.87–40.04)

a Adjusted for child’s age in years, PIR, maternal age at child’s birth, child’s sex, child’s race, prenatal tobacco smoke exposure, cotinine levels, and blood lead levels.

**Table 4:** Poisson regression analysis for CD symptom count in the past year among U.S. children 8–15 years of age.^a^

Variable	No. of cases	Total no.	Adjusted symptom ratio^b^ (95% CI)
Age (years)	68	3,082	1.08 (1.02–1.15)
Sex			
Female	31	1,569	Referent
Male	37	1,513	1.37 (1.06–1.75)
PIR			
> 3.00	11	807	Referent
< 1.00	25	875	1.09 (0.77–1.54)
1.00–1.85	21	712	1.09 (0.67–1.80)
1.85–3.00	8	571	1.11 (0.85–1.44)
Race			
White	19	906	Referent
Mexican	11	928	0.88 (0.67–1.16)
Other Hispanic	3	115	0.74 (0.38–1.45)
Black	31	1,026	1.22 (0.95–1.56)
Other	4	107	1.03 (0.63–1.67)
Maternal age at birth of child (years)			
> 18	54	2,739	Referent
≤ 18	14	343	1.19 (0.80–1.77)
Prenatal tobacco exposure			
No	40	2,577	Referent
Yes	25	470	1.68 (1.14–2.48)
Cotinine level			
First quintile (< 0.015 ng/mL)	3	509	Referent
Second quintile (0.015–0.037 ng/mL)	4	582	0.74 (0.38–1.45)
Third quintile (0.038–0.126 ng/mL)	9	568	1.31 (0.93–1.85)
Fourth quintile (0.127–0.673 ng/mL)	17	622	1.61 (1.04–2.48)
Fifth quintile (0.674–9.9 ng/mL)	26	464	1.97 (1.15–3.40)
Blood lead quartiles			
First quartile (0.2–0.7 μg/dL)	4	655	Referent
Second quartile (0.8–1.0 μg/dL)	11	684	1.55 (1.09–2.22)
Third quartile (1.1–1.4 μg/dL)	22	630	1.50 (1.04–2.17)
Fourth quartile (1.5–10.0 μg/dL)	29	898	1.73 (1.23–2.43)

a Adjusted for child’s age in years, PIR, maternal age at child’s birth, child’s sex, child’s race, prenatal tobacco smoke exposure, cotinine levels, and blood lead levels.

b The symptom ratio is the increase in the symptom rate in the index category compared with the referent category.
